# Unusual course of a Bricker ileal conduit: stenosis from kinking and delayed bowel obstruction

**DOI:** 10.1097/RC9.0000000000000487

**Published:** 2026-04-14

**Authors:** Zhangjie Zheng, Feng Tang, Madanyeti Aers, Tao Liu, Jianping Peng

**Affiliations:** a Department of Urology, Zhongnan Hospital of Wuhan University, Wuhan, China; b Medical Science Research Center, Zhongnan Hospital of Wuhan University, Wuhan, China; c Department of Urology, The Second Affiliated Hospital of Nanchang University, Nanchang, China

**Keywords:** bladder cancer, conduit torsion, hydronephrosis, ileal bladder, urethral reconstruction, urinary diversion

## Abstract

**Introduction:**

Ileal conduit diversion is a common surgical approach following radical cystectomy. This case report describes a rare complication of conduit torsion, which led to the obstruction of the urinary tract and the intestine.

**Case presentation:**

A 71-year-old female developed hydronephrosis after undergoing a Bricker ileostomy. Imaging studies showed that the conduit was narrowed due to torsion. Due to severe adhesions, the surgical correction failed. The patient received chronic stents. Six months later, the patient was readmitted for an intestinal obstruction and required surgical treatment.

**Discussion:**

Conduit torsion is a rare cause of postoperative obstruction. It may be caused by postoperative adhesions. The uniqueness of this case lies in the fact that the same problem simultaneously caused obstruction in both the urinary tract and the intestine. Early surgical intervention is crucial, but due to adhesions, it may present challenges.

**Conclusion:**

The torsion of an ileal conduit may lead to dual obstructions. Once identified, early surgical repair should be considered as soon as possible. Precise surgical procedures are essential for preventing such complications.

## Introduction

Bladder cancer (BLCA) is one of the common malignant tumors in the urinary system. Based on the depth of tumor invasion, BLCA can be roughly classified into two subtypes: muscle-invasive bladder cancer (MIBC) and non-muscle-invasive bladder cancer (NMIBC). For MIBC, radical cystectomy (RC) combined with urinary diversion (UD) remains the standard curative intervention^[^1^]^. Ileal conduit diversion is a frequently employed technique due to its technical simplicity and reliability. Nevertheless, late complications can significantly impair renal function and quality of life. This report details a case of ileal conduit stenosis presenting as dual urinary and intestinal obstruction. We present this case in accordance with the SCARE criteria^[^2^]^ to highlight diagnostic challenges, underscore the importance of early surgical reassessment, and inform preventive strategies.

HIGHTLIGHTS

- This report presents a rare case of urinary and subsequent bowel obstruction caused by torsional stenosis of a Bricker ileal conduit.
- Dense adhesions prevented initial surgical correction, leading to management with chronic ureteral stenting.
- The patient later developed intestinal obstruction requiring surgical intervention, demonstrating the potential for dual complications from a single etiology.
- The case underscores the importance of early surgical revision for conduit torsion and the need for meticulous technique to prevent adhesions.

## Case report

A 71-year-old female presented with bilateral hydronephrosis after undergoing RC with ileal conduit diversion for BLCA. In June 2020, a mass was observed in the bladder during a routine physical examination, leading to an initial transurethral resection. Subsequent pathological analysis revealed high-grade invasive urothelial carcinoma with cancer cells invading the submucosal layer but not the muscular layer [immunohistochemistry: CK7+, CK20+, CD44+, Ki-67 (labeling index: 60%), GATA-3+, P40+]. A surveillance cystoscopy in October 2020 identified tumor recurrence, thus requiring another transurethral resection. The pathology confirmed that the recurrent highly invasive urothelial carcinoma had invaded the lamina propria. The patient subsequently underwent laparoscopic RC with ileal conduit diversion. Final pathology of the cystectomy specimen confirmed high-grade invasive urothelial carcinoma (two tumors, with sizes of 1.1 × 1.0 × 1.0 cm and 1.3 × 0.6 × 0.4 cm respectively), with invasion into the subepithelial connective tissue (closest distance to muscularis propria was less than 0.1 cm). No tumor was found at all surgical margins, including the ureteral stump, and no lymph node metastasis was found in 16 resected lymph nodes (pT1N0MX).

Two months postcystectomy, the patient developed severe hydronephrosis requiring bilateral nephrostomy tube placement. Subsequent ureteroscopy identified stenosis at the level of the ileal conduit. No other abnormalities were found in the medical history. The patient had multiple treatments for hydronephrosis, which was treated by performing ureteral stent placement. Physical examination revealed tenderness at bilateral costovertebral angles, patent nephrostomy sites draining slightly turbid urine, and an erythematous stoma with minimal urinary output.

**Figure 1.**
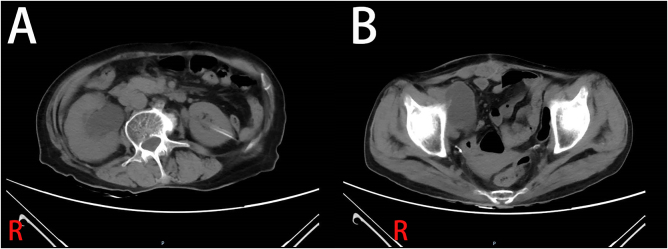
Preoperative CT imaging. (A) Axial view demonstrates significant right-sided hydronephrosis with marked dilation of the renal pelvis and proximal ureter. (B) Coronal view shows the relationship between the ileal conduit and the right pubic bone.

**Figure 2.**
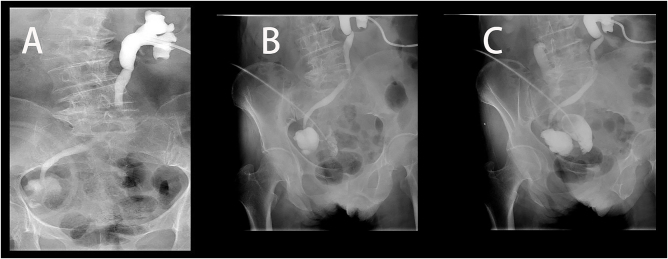
Retrograde contrast studies of the urinary diversion. (A) Antegrade nephrostogram via the left nephrostomy shows a dilated left collecting system and ureter. The contrast could not penetrate the conduit, indicating complete obstruction at the uretero-conduit anastomosis. (B and C) Retrograde conduitogram via the right lower abdominal stoma. Injection of contrast reveals a localized stenosis (arrow in B) within the mid-portion of the ileal conduit, creating a segmental obstruction and separating the opacified proximal and distal limbs. Schematic representation in (C) illustrates the anatomy of the stenotic segment.

Computed tomography (CT) on admission demonstrated significant dilatation of the right renal pelvis and ureter, with abrupt narrowing at its junction with the ileal conduit (Fig. 1). X-ray urography further confirmed a localized stenotic segment within the ileal loop (Fig. 2). Laboratory evaluation revealed acute renal function (blood urea nitrogen: 13.30 mmol/l and creatinine: 185.90 µmol/l), leukocytosis (15.88 × 10^9^/l), a positive urine culture, and elevated procalcitonin (17.89 ng/ml).

Given clinical and radiographic evidence of conduit kinking/torsion, likely attributable to progressive fibrous adhesions, the patient underwent exploratory laparotomy. Intraoperatively, the ileal conduit was found to be densely adherent to the pubic bone, precluding safe mobilization or untwisting. Consequently, definitive surgical correction was abandoned. Instead, a conduit catheter was placed for continuous drainage, and the nephrostomy tubes were retained temporarily before eventual removal in January. Thereafter, the patient relied on chronic ureteral stents; however, residual renal dysfunction and recurrent urinary tract infections persisted.

**Figure 3.**
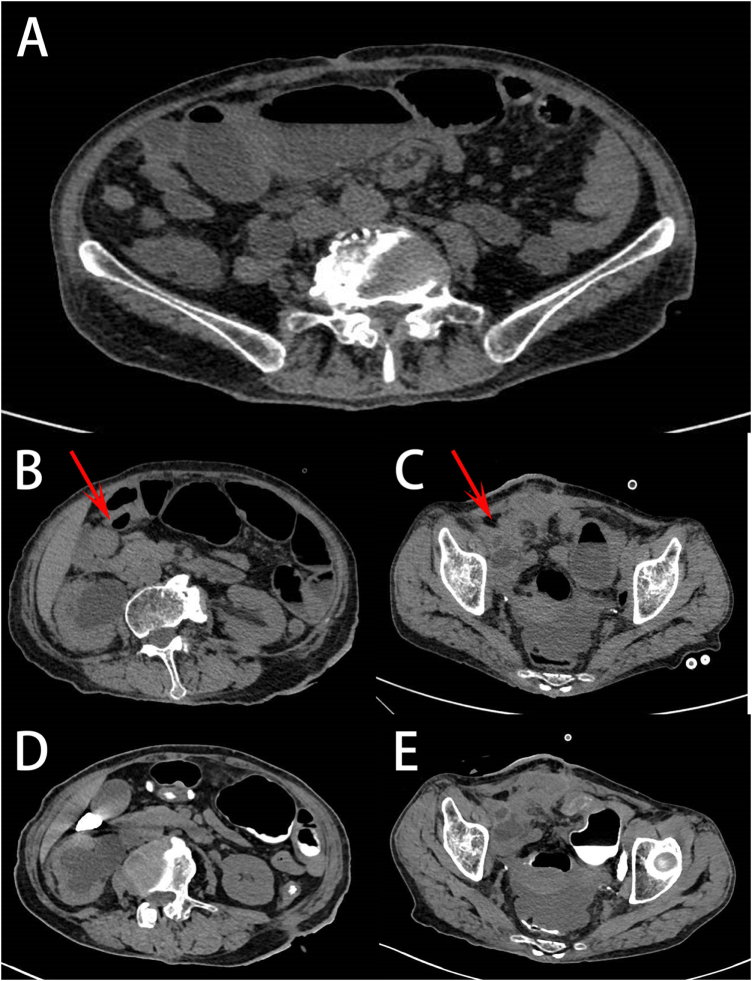
Follow-up CT imaging upon presentation with intestinal obstruction. (A) Emergency CT scan (axial view) reveals air-fluid levels (arrow) within dilated loops of the small intestine, corresponding to mechanical obstruction. (B and C) CT (axial and coronal views, respectively) scan at admission demonstrates the presence of soft tissue density mass (red arrow) in the right lower abdominal wall adjacent to the dilated intestine and the persistence of marked dilation of the right renal pelvis and ureter. (D and E) Contrast-enhanced CT images in axial and coronal planes clarify the obstructive mass and its relationship to surrounding structures.

Six months later, the patient was admitted to the gastrointestinal surgery department with intestinal obstruction. A follow-up CT scan showed soft tissue density mass in the right lower abdominal wall, adjacent dilated small intestine loops, and persistent right-sided hydronephrosis (Fig. 3). A multidisciplinary team diagnosed mechanical small intestine obstruction secondary to chronic conduit torsion and recommended urgent surgical exploration. The surgery was successful in relieving the intestinal obstruction. Unfortunately, the patient was subsequently lost to long-term follow-up.

## Discussion

BLCA imposes a substantial global health burden, with over 430 000 new cases diagnosed annually^[^1^]^. It is clinically and pathologically stratified into MIBC and NMIBC^[^3^]^. For MIBC, the guidelines recommend a combination of treatment modalities such as transurethral resection of bladder (TURBT) and drug instillation in conjunction with the grading of the tumor^[^4,5^]^. RC remains the standard curative intervention for MIBC and high-risk NMIB^[^6–8^]^. Following bladder removal, UD is an obligatory component of RC. Functionally, UD can be classified into continent reservoirs (e.g., continent pouches like Kock, Miami, and Indiana, and orthotopic neobladders) and non-continent reservoirs (e.g., ileal conduit and cutaneous ureterostomy). These complex procedures, which involve intestine manipulation and the creation of multiple anastomoses, inherently associated with significant late complication rates^[^9,10^]^. Complications specific to the ileal conduit include conduit calculi, stomal stenosis, and uretero-enteric anastomotic stricture^[^11^]^.

We report a complex case of urinary obstruction secondary to ileal conduit stenosis, which was rarely documented. The patient ultimately underwent RC with ileal conduit diversion due to recurrent bladder malignancy. Postoperatively, the patient developed persistent upper urinary tract dilatation, which proved refractory to multiple conservative interventions. The etiology was eventually attributed to a torsional stenosis of the ileal conduit. During the initial attempts at surgical correction, extensive dense adhesions rendered a definitive revision unfeasible and prolonged catheter drainage was necessary for decompression. Notably, the patient subsequently re-presented to the gastrointestinal surgery service with a recurrent intestinal obstruction, further complicating the clinical course.

The ileal conduit, pioneered by Bricker in 1950, has been widely adopted and is still considered a highly appreciated method for UD in BLCA^[^8^]^. Although this technique typically obviates the need for permanent ureteral stenting, complications related to intestine harvest and manipulation are frequently reported^[^12^]^. Common complications associated with the Bricker conduit include urinary leakage, enteric leakage, intestinal obstruction, parastomal hernia, and anastomotic stricture, particularly ileoconduit stenosis^[^13–15^]^. The risk of stenotic complications is quite high, and one report indicates that the rate of stenosis resulting from the brick technique (separated and refluxing) is significantly higher than the Wallace technique (conjoined and refluxing)^[^16^]^.

However, primary torsional stenosis of the ileal conduit itself is exceedingly rare. Preventing complications following ileal conduit diversion remains a major postoperative priority. In this case, a series of investigations successfully determined the cause of the urinary obstruction; however, the exact underlying mechanism for the conduit’s deformation and torsion could not be definitively established through subsequent follow-up. We postulate that the torsion may have resulted from adhesive bands either secondary to postoperative scar formation or, less likely, related to local tumor recurrence, which caused tethering and subsequent kinking of the conduit.

This case highlights essential clinical implications. It underscores that conduit torsion is a potential, but relatively rare, complication that can manifest as both urinary tract and intestinal obstruction due to space occupation and involvement in the adhesive process. Early surgical intervention is critical for managing such complications effectively. This experience warrants reflection on strategies to prevent these complex postoperative scenarios, wherein more sophisticated surgical techniques to minimize adhesion formation and more vigilant postoperative surveillance are paramount in mitigating the risk of such secondary obstructions.

## Conclusion

This case illustrates that torsion of an ileal conduit can be a source of both urinary tract obstruction and subsequent intestinal obstruction. It holds significant importance, as it emphasizes the necessity of promptly performing surgical repairs for such complications, even in the face of technical challenges. Refinement of intraoperative practices to mitigate adhesion formation, coupled with structured postoperative surveillance for subclinical conduit dysfunction, represents the pragmatic approach to mitigating the risk of these complex sequelae.

## Data Availability

The data that support the findings of this study are not publicly available due to patient privacy and ethical restrictions, but de-identified data may be available from the corresponding author upon reasonable request and with institutional review board approval.

## Research registration unique identifying number (UIN)

Not appliclable.
